# Comprehensive evaluation of the mechanism of *Gastrodia elata* Blume in ameliorating cerebral ischemia–reperfusion injury based on integrating fecal metabonomics and 16S rDNA sequencing

**DOI:** 10.3389/fcimb.2022.1026627

**Published:** 2022-10-27

**Authors:** Ximeng Ding, Zilu Liu, Yi Liu, Baiyang Xu, Juan Chen, Jingzhe Pu, Deling Wu, Hao Yu, Chuanshan Jin, Xiaoli Wang

**Affiliations:** ^1^ Anhui Province Key Laboratory of Research and Development of Chinese Medicine, Anhui University of Chinese Medicine, Hefei, China; ^2^ Heritage Base of Traditional Chinese Medicine (TCM) Processing Technology of National Administration of Traditional Chinese Medicine (NATCM), Anhui University of Chinese Medicine, Hefei, China; ^3^ Engineering Technology Research Center of Modernized Pharmaceutics, Anhui Education Department Anhui University of Chinese Medicine (ACUM), Hefei, China; ^4^ Anhui Institute for Food and Drug Control, Hefei, China; ^5^ College of Traditional Chinese Medicine, BoZhou University, Bozhou, China; ^6^ Anhui Province Key Laboratory of Traditional Chinese Medicine Decoction Pieces of New Manufacturing Technology, Bozhou, China

**Keywords:** cerebral ischemia–reperfusion injury, *Gastrodia elata* Blume, inflammation, intestinal flora, fecal metabonomics

## Abstract

*Gastrodia elata* Blume was used to treat stroke and headaches caused by “Feng” for thousands of years. The present study has shown a significant effect of *G. elata* Blume in improving cerebral ischemia–reperfusion injury (CIRI). However, the mechanism of *G. elata* Blume in improving CIRI by regulating the intestinal flora has not been reported until now. This research aimed to comprehensively evaluate the mechanism of *G. elata* Blume in CIRI based on fecal metabolomics and 16S rDNA sequencing. The rat model with CIRI was created based on the Zea Longa method. Enzyme-linked immunosorbent assay (ELISA) was used to monitor the inflammatory factors in rat serum. Damages of brain tissues were observed using hematoxylin and eosin (H&E) staining. Cerebral infarction was observed by 2,3,5-triphenyltetrazolium chloride (TTC) staining. The balance of intestinal flora in cecal contents of rats was evaluated by high-throughput sequencing. Changes of metabolites in the intestinal flora were evaluated by fecal metabolomics through Ultra high performance liquid chromatography-orbitrap exploris-mass spectrometer (UHPLC-OE-MS). The area of brain necrosis, cerebral infarction volume, and the contents of inflammatory factors in CIRI rats can be effectively reduced after oral administration of *G. elata* Blume. CIRI can cause disturbances in the intestinal flora and its associated metabolites. *G. elata* Blume can significantly regulate the composition of the intestinal microflora. It reversed CIRI-induced changes in the levels of multiple intestinal bacteria, including *Prevotellaceae*, *Coriobacteriaceae*; *Prevotella*, *Gamma proteobacteria unclassified*, *Barnesiella*, *Escherichia*, *Shigella*; *uncultured Shigella* sp., *Flavonifractor* sp., *Escherichia* sp. *enrichment culture clone NBAR004*, *Veillonella* sp. *R-32*, and *Lactobacillus intestinalis*. The levels of metabolites in cecal contents were disturbed in rats with CIRI, including amino acid, purine, and sphingolipid metabolism. The changes in the level of biomarkers in amino acid metabolism induced by CIRI were significantly reversed after treatment with *G. elata* Blume. Correlation studies show that *Prevotellaceae* was significantly positively correlated with interleukin (IL)-6, and *L. intestinalis* and L-phenylalanine were negatively interrelated to IL-1β. Beta-glycerophosphoric acid was significantly negatively interrelated to high-sensitivity C-reactive protein (hs-CRP). There were significantly negative correlations between L-phenylalanine and *L. intestinalis*, beta-glycerophosphoric acid and *Prevotellaceae. G. elata* Blume protected against CIRI, which may be related to improved intestinal microflora composition and metabolism, resulting in decreased inflammation.

## Introduction

Cerebrovascular diseases are a leading cause of death or disability according to the World Health Organization. Ischemic stroke accounts for 70%–80% of total strokes and is the leading cause of disability worldwide (Yu et al., 2022). Stroke is one of the “four difficult syndromes” in ancient Chinese medicine. Its main clinical manifestations are sudden syncope, hemiplegia, tongue deviation, and numbness. Modern medicine divides it into ischemic stroke and hemorrhagic stroke. It is generally believed that the acute stage of ischemic stroke refers to the onset of less than 2 weeks (Feske, 2021). Cerebral ischemia–reperfusion injury (CIRI) is a significant issue in the clinical treatment of acute ischemic stroke (Shi et al., 2020). The pathological process of CIRI is complicated and is associated with Ca^2+^ overload, mitochondrial injury, inflammation, apoptosis, accumulation of oxygen free radicals, and excess release of excitatory amino acids (Eltzschig and Eckle, 2020). A previous study showed that ischemic stroke can affect a variety of intestinal flora (Zhang P. P. et al., 2021). Dysregulation of intestinal flora may interfere with brain physiological functions and lead to brain diseases. Studies have indicated that brain function could be affected by intestinal flora through the gut–brain axis. The physiological functions of the brain have been shown to be disrupted by metabolites from the intestinal flora such as lipopolysaccharides, neurotransmitters, and short-chain fatty acids (SCFAs) (Cheng et al., 2022).

The intestinal flora plays an important role in metabolism, signal transduction, and immunity. Sequencing of 16S rDNA has become an essential means of studying microorganisms (Yang et al., 2021). Analysis of species composition and diversity is commonly performed by sequencing of microorganisms. This approach allows for a wide detection range, good repeatability, and excellent consistency. A recent study has found that brain diseases could be regulated and controlled by metabolites of the intestinal flora (Li et al., 2018). Metabolomics has been used to evaluate the physiological state of the body because of its integrity (Liu et al., 2022). Metabolomics is necessary to discover the interconnection between intestinal flora metabolites and changes that occur during CIRI to further characterize disease progression and identify disease-related biomarkers (Xian et al., 2022). The research on metabolomics was in possession of dynamic, integral, and systematic characteristics. It was consistent with the holistic view of traditional Chinese medicine (TCM) (Zhang et al., 2019). Liquid chromatography coupled with tandem mass spectrometry (LC-MS) combined with multivariate statistical analysis has become the most powerful tool in the field of metabolomics research. This combination allows for good separation performance and high sensitivity and specificity, which is suitable for qualitative and quantitative analysis (Tang et al., 2022). Compared with gas chromatography and mass spectroscopy (GC-MS), LC-MS does not require complex derivatization of samples and is also suitable for samples with poor thermal stability, poor volatilization, and large molecular weights (Šimková et al., 2022).

**Graphical Abstract:**
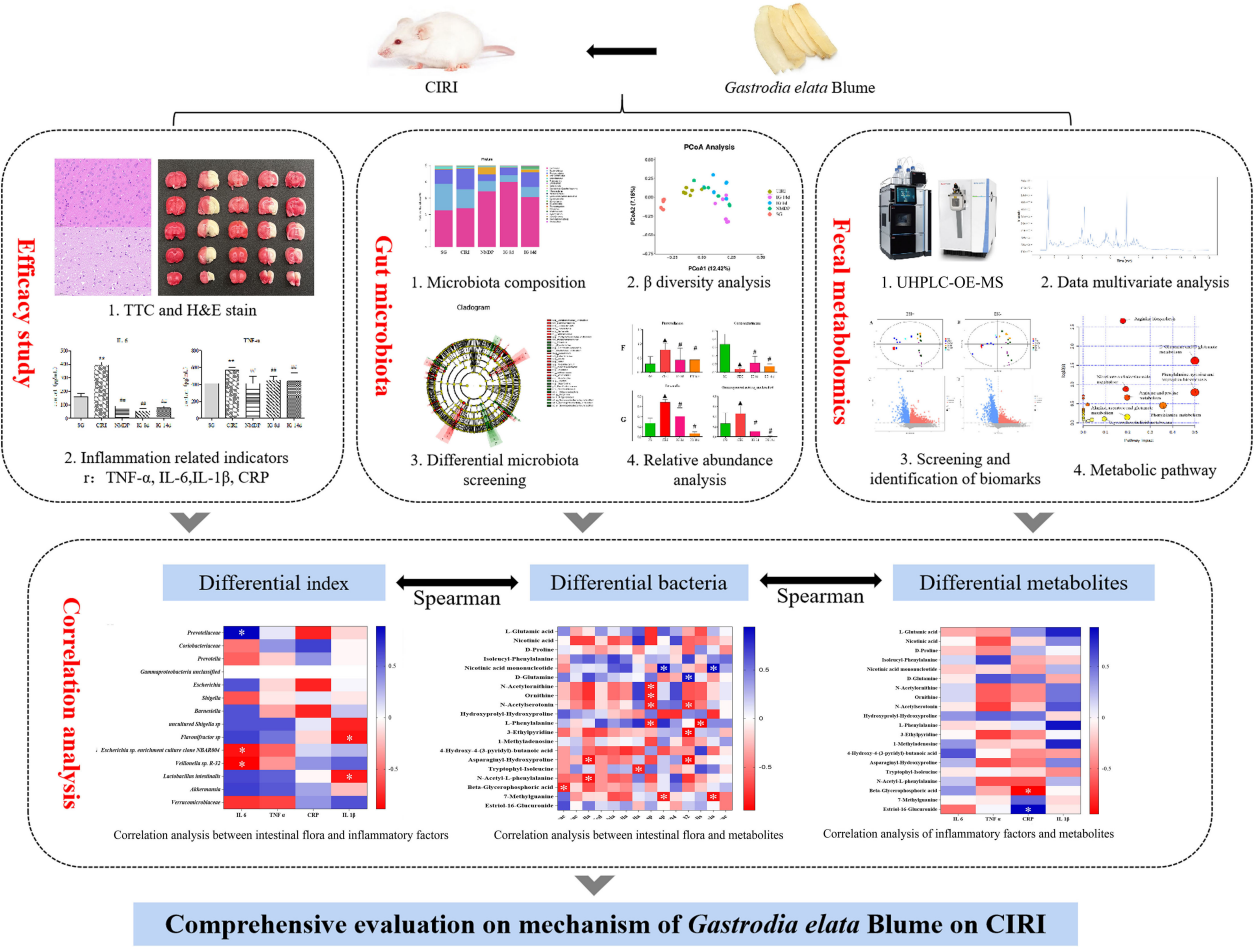


*Gastrodia elata* Blume was first recorded in “Shennong Bencao Jing” listed as a first-class herbal medicine. According to the Chinese Pharmacopoeia, *G. elata* Blume is the dried tuber of *Gastrodia elata* Bl of the orchidaceae plant and induces calming “Feng,” relieving “Jing,” and restraining “Gan Yang.” Ischemic stroke was geared to “stroke” in TCM theory, and *G. elata* Blume is an herb of calming “Feng.” Studies have shown that *G. elata* Blume was an effective treatment for CIRI through antioxidant, anti-inflammatory, antiplatelet aggregation, Ca^2+^ channel blockade, and antiapoptotic effects (Zhang H. S. et al., 2021; Cui and Zhang, 2021). *G. elata* Blume provides unique advantages for the treatment of CIRI, which is associated with complex pathological mechanisms. A study has shown that long-term use of fresh *G. elata* Blume could regulate intestinal flora in rats (Liu F. Y. et al., 2021). However, few studies have evaluated whether the intestinal flora and metabolites of CIRI model rats differed from those of normal rats or whether *G. elata* Blume regulated the intestinal flora in CIRI model rats. In the research, 16S rDNA sequencing combined with metabolomics can characterize the mechanisms by which *G. elata* Blume improved intestinal flora and altered metabolites in rats with CIRI. Therefore, it was of great significance to study the mechanism of *G. elata* Blume with the treatment of CIRI rats by regulation of the intestinal microflora and its metabolites.

This study revealed the correlation between CIRI and intestinal flora by exploring the mechanism of *G. elata* Blume to improve CIRI and provided a new idea for the treatment of CIRI. It also provided a new strategy and direction for the research on TCM in the process of CIRI treatment.

## Materials and methods

### Animal study

Male specific pathogen-free (SPF)-grade Sprague–Dawley (SD) rats were provided by Pizhou Oriental Breeding Co., Ltd. [No. Scxk (Su) 2017-0003]. All experiments were subject to approval by the Committee on the Ethics of Animal Experiments of Anhui University of Chinese Medicine. All of the rats were anesthetized by intraperitoneal injection with pentobarbital (30 mg/kg body weight).

### Chemicals and reagents

*G. elata* Blume (Lot No. 20210812) was purchased from Tongling Hetian Chinese Medicine Group Corporation (Tongling, China) and identified as the dried tuber of the orchidaceae plant *G. elata* Bl by Professor Liu Shoujing (Anhui University of Traditional Chinese Medicine). Nimodipine tablets (Lot No. 210126) were provided by Yabao Pharmaceutical Co., Ltd. (Shanghai, China), and gentamycin sulfate injection (Lot No. 2005080121 2-12) was purchased from Xiandai Hasen Pharmaceutical Co., Ltd. (Shanghai, China). 2,3,5-Triphenyltetrazolium chloride (TTC, Sigma™, USA). UHPLC system (Vanquish, Thermo Fisher Scientific), UHPLC BEH Amide column (Waters, 2.1 mm × 100 mm, 1.7 μm); Mass Spectrometry (Orbitrap Exploris 120, Thermo Fisher Scientific).

### Preparation of water extracts of *Gastrodia elata* Blume and nimodipine solution

One hundred grams of sample was weighed into a beaker, and 1,000 ml of water was added. After standing for 30 min, it was decocted for 30 min and then filtered. This process was repeated, and the filtrates were merged and concentrated to 0.81 g/ml. Nimodipine tablets were ground into powder and prepared at a concentration of 0.9 mg/ml. All water extracts were shaken well before using.

### Animals and drug administration

At the same time of the experiment, the dosage of *G. elata* Blume was also preliminarily screened, and it was found that the high, middle, and low doses had a significant effect on the volume ratio of cerebral infarction (**Supplementary Figure 1**). In this experiment, the medium dose is selected for administration. The medium dose refers to the dose of rats corresponding to the commonly used dose in humans.

After a week of adaptive feeding, 60 male SPF-grade SD rats were modeled by the Zea Longa method. An incision was made in the midline of the neck region, and the common carotid artery and left external carotid artery were isolated and ligated. The internal carotid artery was inserted with a 0.26-mm fishing line. After 2 h of occlusion, a certain length of fishing line was pulled out; reperfusion was allowed to occur for 24 h. The modeled rats were scored for signs of neurological impairment by the 5-point Zea Longa method (Longa et al., 1989): 0 point, normal; 1 point, weakness of the left forelimb and incomplete extension; 2 points, turning to the left side when walking; 3 points, cannot bear weight on the left side; and 4 points, no spontaneous activity and disturbed consciousness. Those rats with scores of 2–3 were included in the successful model.

Twenty-four male SPF-grade SD rats (220 ± 20 g) were randomly separated into the model group (CIRI), oral administration of nimodipine (NMDP) group, intragastric administration of *G. elata* Blume for 8 days (IG 8d) group, and intragastric administration of *G. elata* Blume for 14 days (IG 14d) group. Six rats were in the sham group (SG). The SG was subjected to the surgical procedure without undergoing carotid occlusion. All other groups underwent carotid occlusion. The rats were orally administered physiological saline, nimodipine solution for 14 days (9 mg/kg), or water extracts of *G. elata* Blume for 8 and 14 days (8.1 g/kg).

### Collection and preparation of samples

#### Collection of samples

On the 7th and 13th days after administration of *G. elata* Blume, rats were fasted but allowed access to water. Pentobarbital (30 mg/kg body weight) was injected intraperitoneally to anesthetize the rats on the next day. Blood was collected from the abdominal aorta into the negative-pressure tube. Then, it was separated at 3,500 rpm and 4°C lasting for 15 min. Supernatant was aspirated, then placed at -80°C. Take out the brain, wash it, and absorb the water, then put it in 4 % paraformaldehyde (Lot No. 2004A08, Zhenwo Biomedical Technology Co., Ltd. Hefei, China). The cecum contents were removed, quickly frozen, and stored at -80°C.

#### Sample preparation of the cecum

Weigh 25 mg of sample and add 500 μl of solution (methanol:acetonitrile:water = 2:2:1, isotopically labeled internal standard mixture). Intestinal contents were ground evenly for 4 min at 35 Hz and underwent ultrasonic treatment in bath with ice water lasting for 5 min. Pestling and ultrasonic cycle three times. Then, the incubated at -40°C for 1 h and 15 min of separation at 12,000 rpm and 4°C. Take supernatant. Sample of quality control (QC) was mixed by all of the samples.

### Experimental methods

#### 2,3,5-triphenyltetrazolium chloride (TTC) staining

The brain tissue was cut horizontally into five pieces, placed in 5% TTC solution, incubated at 37°C for 15 min, then put into 10% paraformaldehyde. Pale staining indicates the infarct area; the infarct volume of the rat brain tissue and the volume of the whole brain tissue were quantitatively analyzed by ImageJ software. Infarct ratio (%) = infarct volume/whole brain volume × 100%.

#### Hematoxylin and eosin staining

The brain tissue that was stored in 4% paraformaldehyde was embedded in paraffin, cut into 5-μm-thick sections, and stained with hematoxylin and eosin (H&E). Morphological changes in the brain tissues were observed.

#### Detection of biochemical indices in rat serum

Enzyme-linked immunosorbent assay (ELISA) (Jianglaibio) was used to test the content of tumor necrosis factor-α (TNF-α), interleukin-1β (IL-1β), interleukin-6 (IL-6), and high-sensitivity C-reactive protein (hs-CRP) in serum according to the manufacturer’s instructions. An automatic enzyme immunoassay analyzer (Multiskan Spectrum) was used for detection.

#### Sequencing and analysis of intestinal flora

DNA from different samples was extracted using the E.Z.N.A. ®Stool DNA Kit (D4015, Omega, Inc., USA) then eluted in 50 μl elution buffer, stored at -80°C. Blank is nuclease-free water. The 5’ ends of the primers were tagged with specific barcodes per sample and sequencing universal primers. PCR conditions for amplification of prokaryotic 16S fragment are as follows: reaction at 98°C for 30 s, 98°C for 10 s and repeated 32 times, 54°C for 30 s, and 72°C for 45 s, and final reaction at 72°C for 10 min. In this study, 2% agarose electrophoresis was used to detect the purity. The control was ultrapure water. Non-target products were excluded using AM Pure XT XP beads (Beckman Coulter Genomics, Danvers, MA, USA) and quantified by Qubit (Invitrogen, USA). The amplicon pools were prepared for sequencing, and the average molecular length was measured with the Agilent 2100 Bioanalyzer (Agilent, USA). Sequencing was performed using the NovaSeq PE250 platform of Illumina (Kapa Biosciences, Woburn, MA, USA). Primer information is shown in **Table 1**.

**Table 1:** Amplified primer information.

Region	Primers
V3-V4	341F (5'-CCTACGGGNGGCWGCAG-3') 805R (5'-GACTACHVGGGTATCTAATCC-3')
V4	515F(5'-GTGYCAGCMGCCGCGGTAA-3') 806R (5'- GGACTACHVGGGTWTCTAAT-3')
V4-V5	F(5’-GTGCCAGCMGCCGCGG-3’) R(5’-CCGTCAATTCMTTTRAGTTT-3’)
Archae	F(5’-GYGCASCAGKCGMGAAW-3’) R(5’-GGACTACHVGGGTWTCTAAT-3’)

### Conditions of chromatography and mass spectrometry

#### Chromatographic conditions

The UHPLC system featured a UHPLC BEH Amide column (2.1 mm × 100 mm, 1.7 μm) coupled to an Orbitrap Exploris 120 mass spectrometer. The mobile phase: 25 mmol/L ammonium acetate and 25 ammonia hydroxide in water (pH = 9.75) (A) and acetonitrile (B). The gradient program: 0~0.5 min, 95% B; 0.5~7 min, 95%~65% B; 7~8 min, 65%~40% B; 8~9 min, 40% B; 9~9.1 min, 40%~95% B; 9.1~12 min, 95% B. Flow rate: 0.5 ml/min, column temperature: 30°C, autosampler temperature: 4°C, injection volume: 2 μl.

#### Mass spectrometer conditions

Sheath gas flow rate: 50 Arb, Aux gas flow rate: 15 Arb, capillary temperature: 320°C, full MS resolution: 60,000, MS/MS resolution: 15,000, collision energy: 10/30/60 in negative mode, and spray voltage: 3.8 kV (positive) or -3.4 kV (negative).

### Data and statistical analysis

#### 16S rDNA data processing

Quality filtering was done by fqtrim (v0.94). Chimeric sequences were filtered by Vsearch software (v2.3.4). Dereplication was done using DADA2. Feature abundance was normalized by SILVA (release 132) classifier. Indices, β-diversity were computed by QIIME2. Sequence was aligned by Blast; feature sequences were annotated by SILVA database.

#### UHPLC-OE-MS data processing

ProteoWizard transformed raw data to mz XML format. An in-house MS2 database (Biotree DB) was used for metabolite annotation. The cutoff for annotation was set at 0.3.

All values are expressed as the mean ± standard error. Statistical significance was defined as *P* < 0.05. Differences in each variable among the three groups were tested using one-way analysis of variance (ANOVA). For non-parametric tests, statistical significance between the three groups was assessed using a two-tailed Mann–Whitney test.

#### Spearman correlation analysis

The Spearman correlation coefficient was used to show the relationship between the parameters, and the correlation coefficient was always between -1 and +1. If the absolute value of the correlation coefficient was closer to 1, the better the linear relationship. In this experiment, Spearman correlation coefficients r > 0.8 and r < -0.8 were used to indicate significant positive and significant negative correlations, respectively.

**FIGURE 1:**
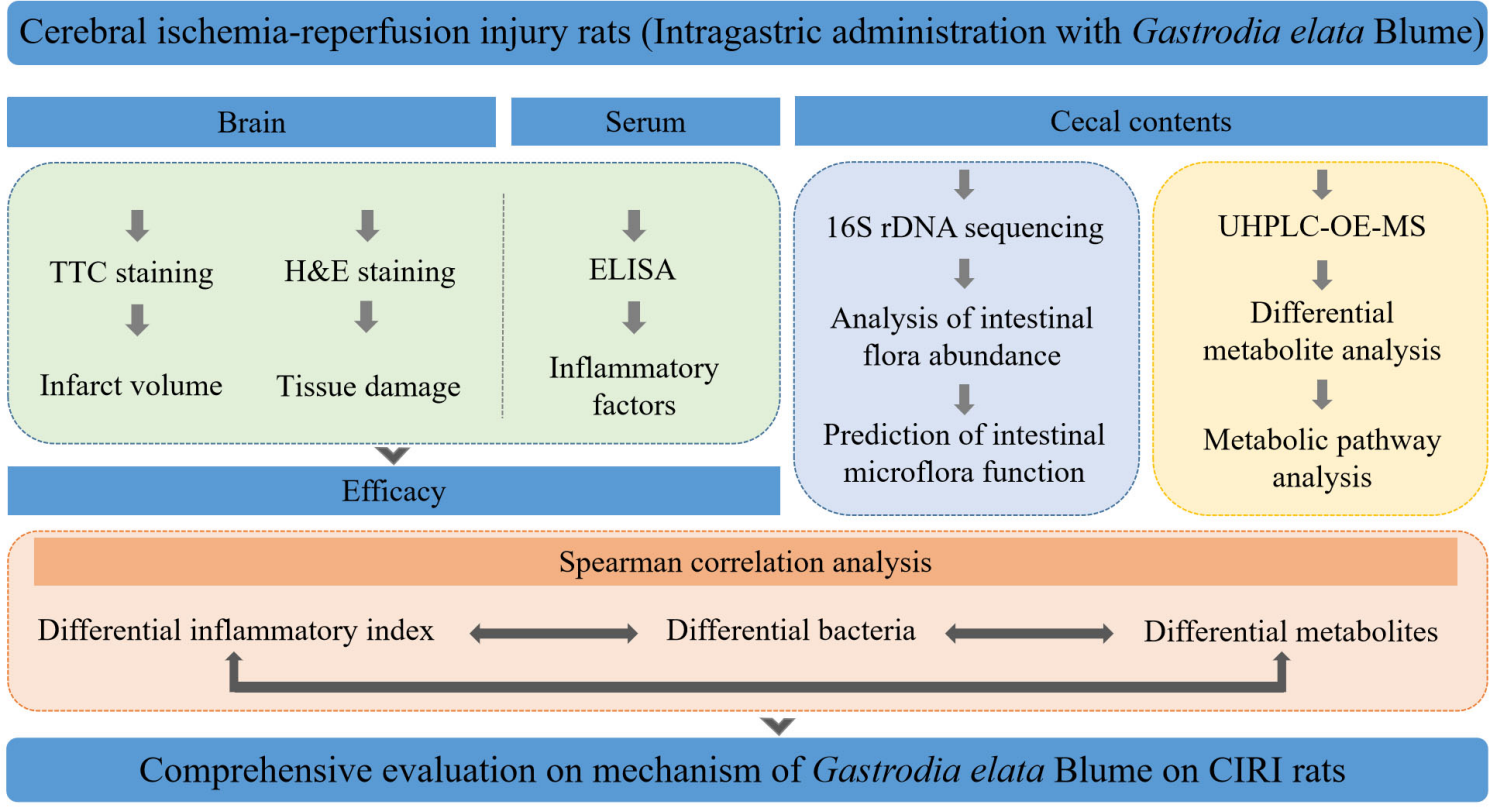
The strategy of this work to evaluate the mechanism of *Gastrodia elata* Blume in ameliorating cerebral ischemia–reperfusion injury.

The deliberate strategy of this work was laid out just as shown in **Figure 1**.

## Results

### Pharmacodynamic evaluation

#### Reduction of the volume of cerebral infarction

**Figure 2:**
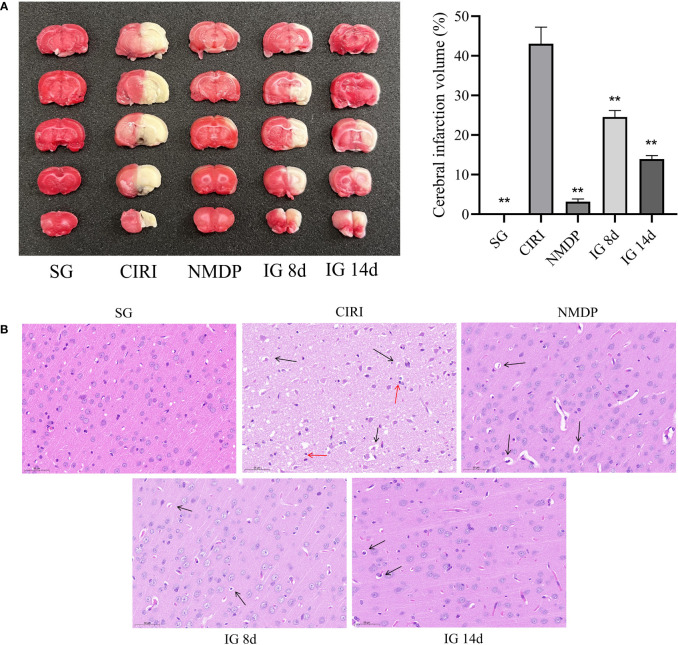
**(A)** Images of the brain by 2,3,5-triphenyltetrazolium chloride (TTC) staining (left). Quantified data of TTC staining presented in ischemia volume (%) (right). **P < 0.01. **(B)** Brain tissue sections of rats in each group. Black arrows mean vacuoles, and red arrows mean inflammatory cell infiltration.

TTC staining of brain tissue sections showed that IG 8d and IG 14d dependently reduced the infarct area compared with CIRI (**Figure 2A**). At the same time, the infarct volume of IG 14d was significantly lower than that of IG 8d. It shows that *G. elata* Blume can significantly reduce the volume of cerebral infarction.

### Pathological observation of brain tissues

The brain tissue sections in the SG were normal (cell structure was complete, cells were in neat rows, the nucleus was in the middle of the cells, and the nucleolus was clear) (**Figure 2B**). Large necrotic areas were observed in the ischemic brain tissue in the model group. The cell structure was unclear, and pyknosis, vacuole formation, and inflammatory cell infiltration were observed. Compared with CIRI, the pathological characteristics and morphology of the cerebral cortex tissue were improved to varying degrees in each treatment group, as evidenced by reduced focal necrosis and inflammatory cell infiltration and more orderly cell arrangement.

### Levels of inflammatory mediators in rat serum

**Figure 3:**
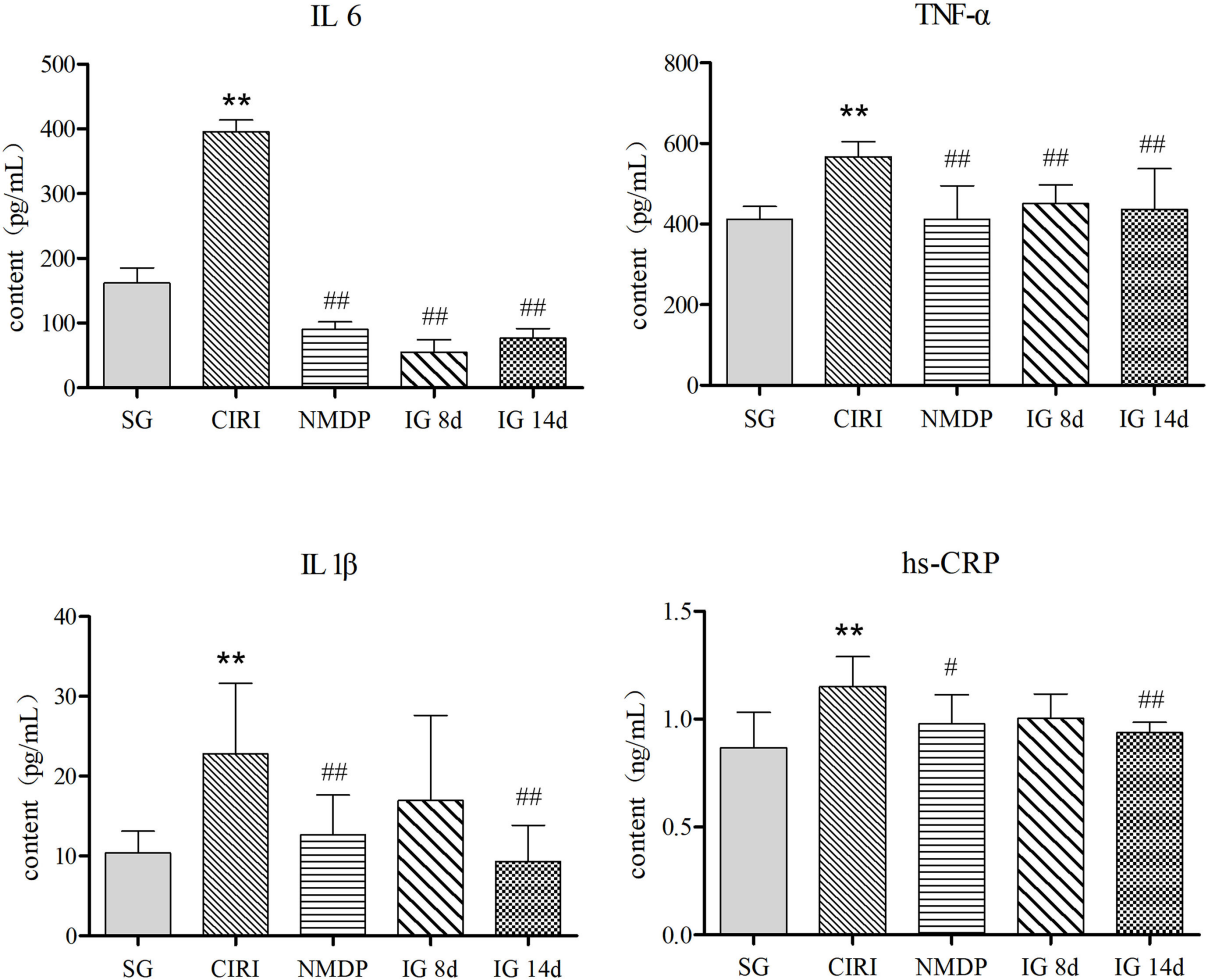
Inflammatory factor content in the serum. Compared with the SG, *P < 0.05, **P < 0.01; compared with the CIRI group, *^#^P* < 0.05, *^##^P* < 0.01.

Compared with the SG (**Table 2**; **Figure 3**), the contents of TNF-α, IL-1β, IL-6, and hs-CRP were conspicuously higher in the CIRI group. In contrast to the CIRI group, the contents of TNF-α and IL-6 in the IG 8d, IG 14d, and NMDP groups were conspicuously decreased. The expression contents of IL-1β and hs-CRP in the IG 14d and NMDP groups were conspicuously lower than those in the CIRI group. The expression contents of IL-1β and hs-CRP in the serum of rats were lower in the IG 8d group than those in the CIRI group but non-conspicuously different (*P* > 0.05).

**Table 2:** Inflammatory factor content in serum.

Group	IL-6 (pg/mL)	TNF-α (pg/mL)	IL 1β (pg/mL)	hs-CRP (ng/mL)
SG	161.67 ± 23.45	412.06 ± 31.61	10.38 ± 2.72	0.87 ± 0.16
CIRI	395.43 ± 18.48^**^	567.17 ± 37.14^**^	22.79 ± 8.86^**^	1.15 ± 0.14^**^
NMDP	90.14 ± 11.55^##^	412.4 ± 82.75^##^	12.65 ± 4.98^##^	0.98 ± 0.14^#^
IG 8d	54.86 ± 19.48^##^	451.19 ± 45.93^##^	16.96 ± 10.67	1.00 ± 0.11
IG 14d	76.63 ± 14.63^##^	436.06 ± 101.64^##^	9.31 ± 4.56^##^	0.94 ± 0.05^##^

Compared with the SG, *P < 0.05, **P < 0.01; compared with the CIRI, ^#^ *P* < 0.05, ^##^ *P* < 0.01.

## *Gastrodia elata* Blume normalized the intestinal flora composition in CIRI Rats

### Species diversity of alpha in samples

**Figure 4:**
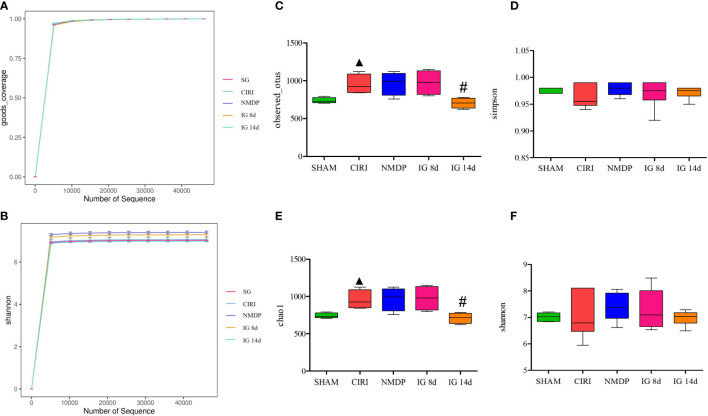
Effect of *Gastrodia elata* Blume on the alpha diversity of the intestinal flora. Note: Analysis of the gut microbial diversity was performed on the basis of 16S rDNA sequencing. Good’s coverage index **(A)** and Shannon index **(B)** are presented. OTUs **(C)**, Simpson index **(D)**, Chao1 index **(E)**, and Shannon index **(F)** were used to describe the alpha diversity of the gut bacterial assemblages in the rats receiving different treatments. ^▲^ *P* < 0.05 when compared with the sham groups, ^#^ *P* < 0.05 when compared with the model groups.

Analysis (**Figure 4**) showed that the depth of sequencing was sufficient, and Operational taxonomic units (OTU) coverage rate was greater than 99% in each experimental group, indicating that the sample assay met the standards of sequencing. In contrast to the SG, alpha diversity of CIRI was increased, which was reversed after 14 days of *G. elata* Blume administration.

### PCoA results

**Figure 5:**
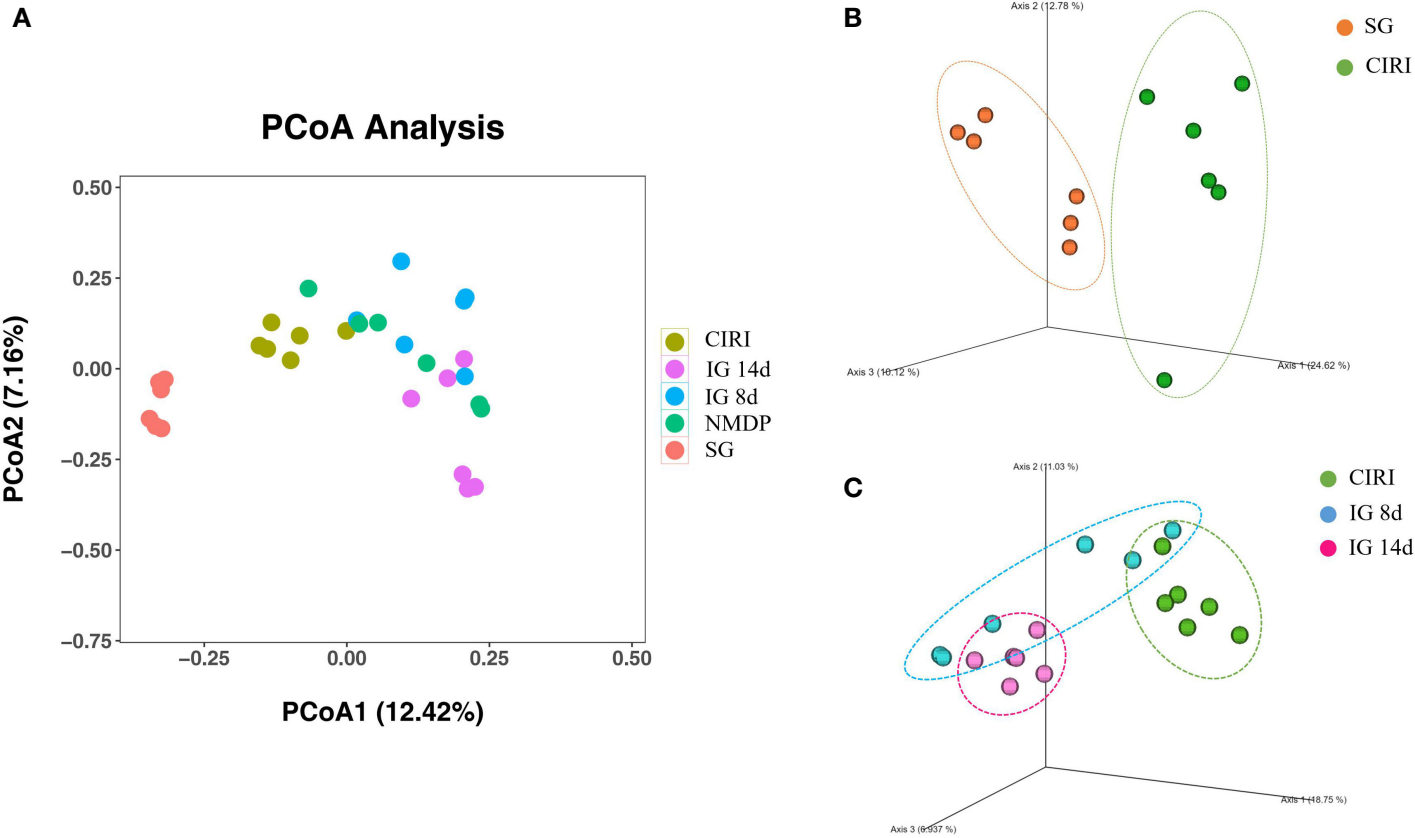
Score of PCoA **(A)**, PCoA (3D) of SG and CIRI **(B)** and PCoA (3D) of CIRI, IG 8d, IG14d **(C)**.

Weighted Principal co-ordinates analysis (PCoA) analysis results (**Figure 5**) showed that the SG and CIRI group could be separated according to intestinal flora composition. It was observed that there were differences among IG 8d, IG 14d, and CIRI groups, and the separation increased evidently with longer courses of IG treatment. It indicated that *G. elata* Blume could regulate the intestinal flora of CIRI rats in a time-dependent manner. With the prolongation of treatment time, the regulation effect on intestinal flora became more obvious.

### Changes in intestinal flora abundance

**Figure 6:**
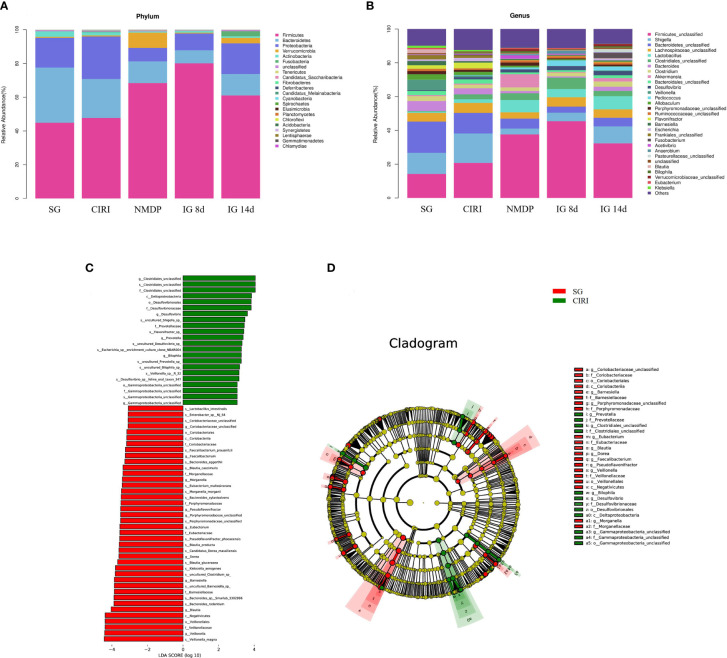
Histogram of the intestinal flora and differential bacteria between the SG rats and CIRI rats. **(A)** Histogram of the intestinal flora at the phylum level. **(B)** Histogram of the intestinal flora at the genus level. **(C)** The vertical coordinate is the classification unit with a significant difference between groups, and the horizontal coordinate shows the logarithm score value of LDA difference analysis of corresponding classification unit intuitively with bar chart and sorts according to the score value, so as to describe the difference in their sizes in different group samples. The longer the length is, the more significant the difference is. **(D)** The hierarchical tree shows the hierarchical relationship of all taxa in the sample community from door to genus (from inner circle to outer circle in turn). The node size corresponds to the average relative abundance of the taxon. The yellow node represents the taxon that does not show significant differences between groups, while other colors (such as green and red) indicate that these taxa show significant group differences, and the abundance is higher in the group sample represented by the color. The letters identify the names of taxa with significant differences between groups.

The intestinal flora of each group of rats included *Firmicutes*, *Bacteroides*, *Actinobacteria*, *Proteobacteria*, *Verrucomicrobia*, *Tenerictes*, *Fusobacteria*, *Patesicibacteria*, *Cyanobacteria*, and *Elusimicrobia*, and the relative levels of *Firmicutes* and *Bacteroides* were highest, accounting for 80% of the total intestinal flora at the phylum level (**Figure 6A**). Although the species of the intestinal flora in all groups are relatively consistent, the abundance of species was variant. In contrast to that of the SG, the abundance of *Proteobacteria* in the CIRI group was conspicuously higher (*Proteobacteria*: 17.63% abundance in the SG, 25.21% abundance in CIRI, *P* < 0.01). At the gene level (**Figure 6B**), *Shigella* in the intestinal microorganisms of the CIRI group was conspicuously higher than that of the SG. *Proteobacteria* and *Shigella* in the NMDP, IG 8d, and IG 14d groups showed a trend toward a return to levels observed in the SG.

### Screening of CIRI-related bacteria and regulation of different bacteria by *Gastrodia elata* Blume

**FIGURE 7:**
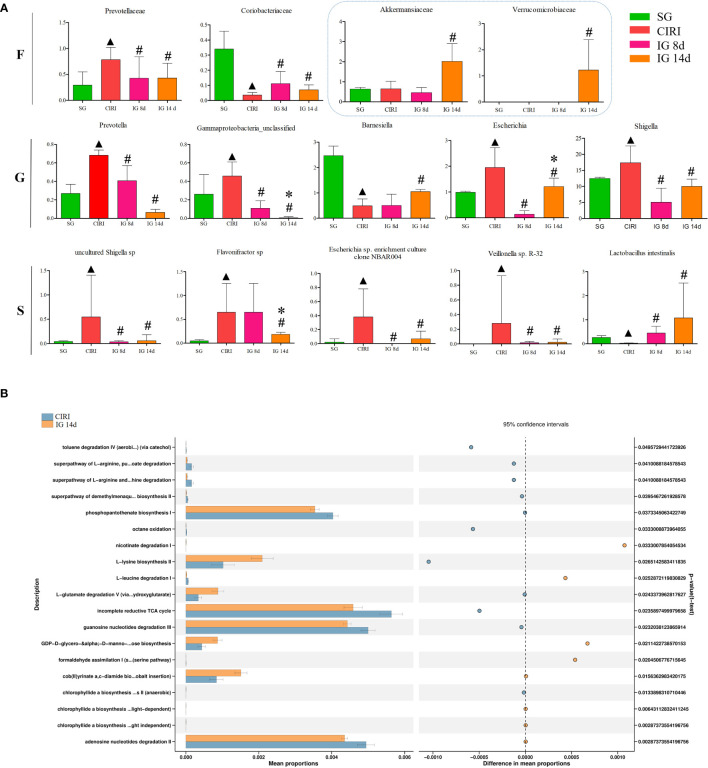
Differential bacteria and histogram of the KEGG gene function difference analysis related to CIRI after administration. Note: N = 6 rats per group, values are presented as mean ± SEM. ^▲^ *P* < 0.05 when compared with the SG, ^#^ *P* < 0.05 when compared with the CIRI, *P < 0.05 when compared with the IG 8d in **(A)**. There is the proportion of differences in functional abundance within the CIRI and the IG 14d, shown in the middle is the percentage of difference in functional abundance within the 95% confidence interval in **(B)**.

To identify bacteria related to CIRI, Linear discriminant analysis Effect Size (LEfSe) software was used to analyze differences between groups (**Figures 6C, D**). Bacteria groups (genus level) with Linear Discriminant Analysis (LDA) >2.0 and *P* < 0.05 were considered conspicuously associated with CIRI. Twenty microbial markers were identified, including the following 12 groups of bacteria: *Prevotellaceae*, *Coriobacteriaceae*; *Prevotella*, *Gamma proteobacteria unclassified*, *Barnesiella*, *Escherichia*, *Shigella*; *uncultured Shigella* sp., *Flavonifractor* sp., *Escherichia* sp. *enrichment culture clone NBAR004*, *Veillonella* sp. *R-32*, and *Lactobacillus intestinalis.* These groups differed significantly between the CIRI group and the groups administered *G. elata* Blume (**Figure 7**), which more closely resembled the SG. Although there were no conspicuous differences in the abundance of *Verrucomicrobiaceae* and *Akkermansiaceae* between the SG and CIRI groups, the levels of *Verrucomicrobiaceae* and *Akkermansiaceae* were conspicuously increased in IG 8d and IG 14d in contrast to those in the CIRI group.

### Analysis of differences in metabolic pathways using Kyoto Encyclopedia of Genes and Genomes analysis

Administration of *G. elata* Blume to CIRI rats resulted in significant changes in gene expression in the intestinal flora (**Figure 7**). *G. elata* Blume may inhibit the degradation of L-arginine and L-orniacin, nicotinic acid I, adenosine nucleotide, and L-leucine in CIRI model rats and increase the degradation of L-glutamate. These results suggested that structural changes in the intestinal flora after *G. elata* Blume administration may lead to changes in its own amino acid metabolism.

### Regulation of metabolites of the intestinal flora of CIRI rats treated with *Gastrodia elata* Blume

#### Quality control

Sample analysis may be affected by various factors, resulting in response signals at the time of detection. QC samples can play a role in confirming the performance of experimental methods and system stability. There are also errors associated with the operator of the instrument. Therefore, QC of data processing is also necessary. Theoretically, all QC samples should be the same, but errors in the experiment may lead to differences between QC samples. QC samples were situated within ±2 SD (**Supplementary Figure 2**) of each other, which indicated that the data quality in this study was excellent. Correlation of QC samples ≥0.7 indicates better method stability and better data quality. The correlation among the QC samples was very high, which indicated that this study had good data quality (**Supplementary Figure 3**).

### Multivariate statistical analysis of metabolites

**Figure 8:**
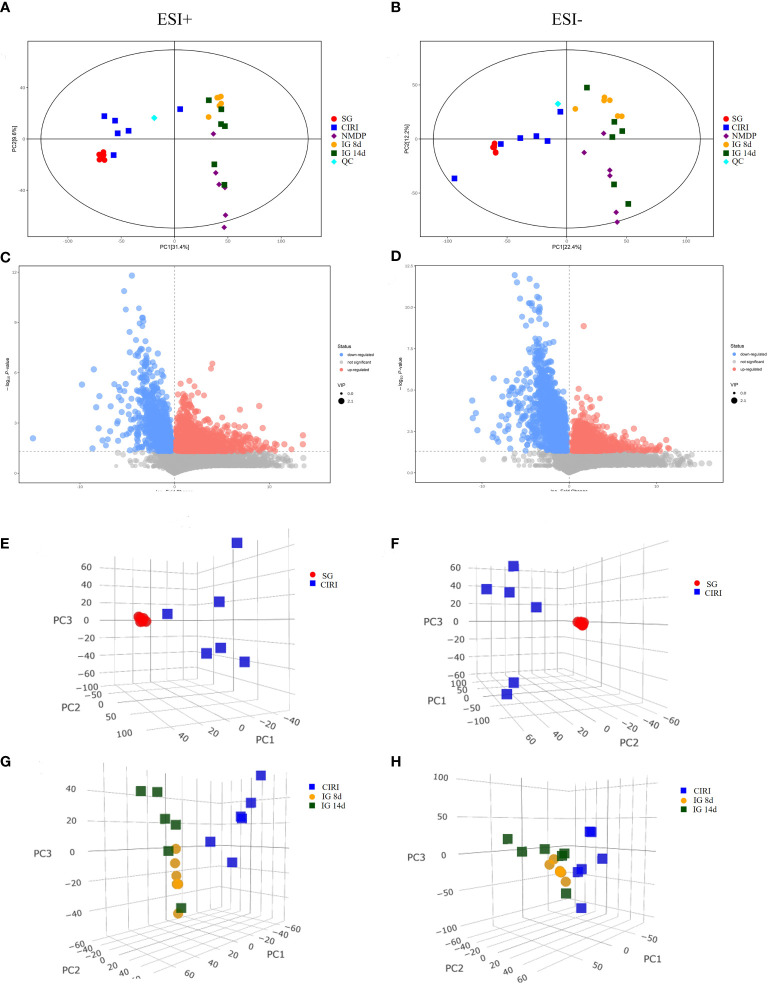
PCA score plot (2D and 3D) and volcano plot of the SG and CIRI group in positive and negative mode. Note: PCA score plot (2D) in positive **(A)** and negative **(B)** mode. Volcano plot of the SG and CIRI group in positive **(C)** and negative **(D)** mode. PCA score plot (3D) of the SG and CIRI group in positive mode **(E)** and in negative mode **(F)**. PCA score plot (3D) of CIRI, IG 8d, and IG 14d groups in positive mode **(G)** and in negative mode **(H)**.

**Figure 9:**
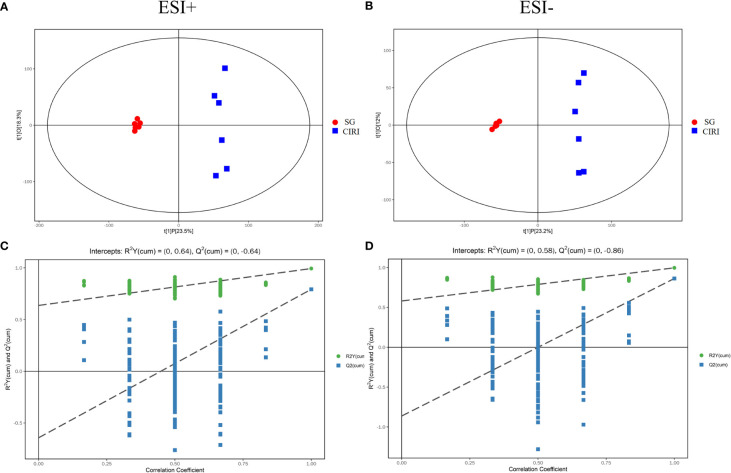
The OPLS-DA score plot **(A)** and OPLS-DA permutation plot **(C)** in positive mode. The OPLS-DA score plot **(B)** and OPLS-DA permutation plot **(D)** in negative mode.

Principal component analysis (PCA) and orthogonal projection to latent structures squares-discriminant analysis (OPLS-DA) models were established based on metabolomics data collected using UHPLC-OE-MS in positive and negative ion modes. The PCA score plot (**Figures 8A, B**), volcano plot (**Figures 8C, D**), and OPLS-DA score plot (**Figures 9A, B**) showed conspicuous differences in CIRI in contrast to the SG, indicating that the metabolic profiles were altered by CIRI. The OPLS-DA model had *R^2^* = 0.64, *Q^2^* = -0.64 in positive ion mode and *R^2^* = 0.58, *Q^2^* = -0.86 in negative ion mode (**Figures 9C, D**). There was no overfitting phenomenon, the model was predictive, and the quality of the model was good. The importance of variables was determined using variable importance in the projection (VIP) by OPLS-DA analysis with the threshold set to 1.0. Metabolic differences among the SG, CIRI, NMDP, IG 8d, IG 14d, and QC groups have been evaluated. As shown in the PCA score plot [three dimensional (3D)] (**Figures 8E–H**), the metabolomic profiles of the IG 8d and IG 14d groups differed significantly from that of the CIRI group, and the IG 14d group metabolomic profile differed most from the CIRI group. With the prolongation of treatment time, *G. elata* Blume has a more obvious regulatory effect on intestinal metabolites.

### Potential biomarkers

**FIGURE 10:**
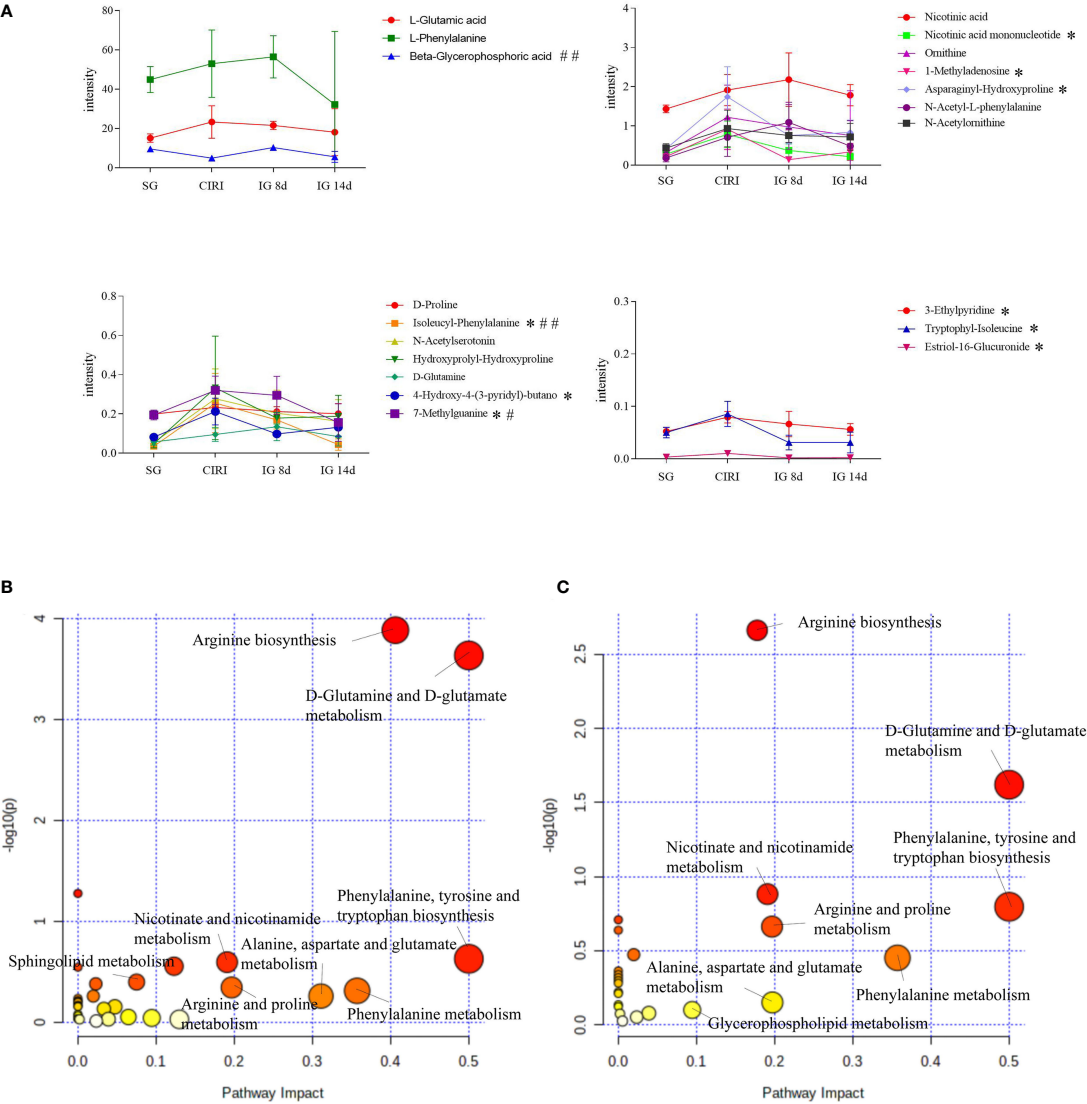
Expression levels of differentially expressed metabolites in the serum in the SG, CIRI, IG 8d, and IG 14d groups **(A)** and affected metabolic pathways. Diagram of the abnormal metabolic pathways in CIRI rats **(B)**. Diagram of the metabolic pathways of *Gastrodia elata* Blume callback **(C)**. *P < 0.05 when IG 14d was compared with the CIRI; *^#^P* < 0.05, *^##^P* < 0.01when IG 14d was compared with the IG 8d in **(A)**.

Metabolomic results showed that endogenous metabolites were altered by CIRI in rats. The combination of VIP >1, *P* < 0.05 was conducted as a standard to search for potential biomarkers. Twenty metabolites in the CIRI and SG groups were associated with CIRI. Administration of *G. elata* Blume reversed most of the differences in metabolite levels observed between the SG and CIRI group. Twenty potential biomarkers were found to distinguish between the SG and CIRI, IG 8d and CIRI, and IG 14d and CIRI (**Table 3**; **Figure 10A**). These potential biomarkers are mainly related to amino acid metabolism. After 14 days of *G. elata* Blume administration, the levels of most amino acids in the cecum contents returned to levels similar to those in the SG, which indicated that *G. elata* Blume protects against CIRI *via* amino acid metabolism.

**Table 3:** Results of potential biomarkers detected in rat cecal contents.

No.	Name	Adduct	Formula	MS2 score(%)	RT(s)	m/z (Da)	Trend (CIRI vs SG)	Trend (IG 8d vs CIRI)	Trend (IG 14d vs CIRI)
1	Nicotinic acid	ESI+	C_6_H_5_NO_2_	0.9978	233.1765	124.038452	↑*	↑	↓
2	D-Proline	ESI+	C_5_H_9_NO_2_	0.9967	622.353	116.0697098	↑*	↓	↓
3	Isoleucyl-Phenylalanine	ESI+	C_15_H_22_N_2_O_3_	0.8371	236.192	279.1681468	↑*	↓*	↓*
4	Nicotinic acid mononucleotide	ESI+	C_11_H_15_NO_9_P	0.8128	353.331	256.0798356	↑*	↓*	↓*
5	D-Glutamine	ESI+	C_5_H_10_N_2_O_3_	0.7855	446.658	147.0753709	↑*	↑	↓
6	N-Acetylornithine	ESI+	C_7_H_14_N_2_O_3_	0.7652	402.323	175.1063854	↑*	↑	↓
7	Ornithine	ESI+	C_5_H_12_N_2_O_2_	0.7638	531.337	133.0960855	↑*	↑	↓
8	L-Glutamic acid	ESI+	C_5_H_9_NO_4_	0.8866	413.4785	148.0591418	↑*	↓	↓
9	N-Acetylserotonin	ESI+	C_12_H_14_N_2_O_2_	0.4674	47.4162	219.1113067	↑*	↓	↓
10	N-Acetyl-L-phenylalanine	ESI-	C_11_H_13_NO_3_	0.7762	193.981	206.0823623	↑*	↑	↓
11	Hydroxyprolyl-Hydroxyproline	ESI+	C_10_H_16_N_2_O_5_	0.7306	252.026	245.1113078	↑*	↓	↓
12	Beta-Glycerophosphoric acid	ESI-	C_3_H_9_O_6_P	0.7878	178.476	171.0081811	↓**	↑	↑
13	L-Phenylalanine	ESI+	C_9_H_11_NO_2_	0.9888	275.8235	166.0846645	↑*	↓	↓
14	3-Ethylpyridine	ESI+	C_7_H_9_N	0.9911	686.931	108.0797709	↑**	↓*	↓*
15	1-Methyladenosine	ESI+	C_11_H_15_N_5_O_4_	0.9882	267.2615	282.1177131	↑*	↓*	↓*
16	4-Hydroxy-4-(3-pyridyl)-butanoic acid	ESI+	C_9_H_11_NO_3_	0.9196	68.10895	182.0797806	↑**	↓*	↓*
17	Asparaginyl-Hydroxyproline	ESI+	C_9_H_15_N_3_O_5_	0.8890	361.207	246.1062813	↑**	↓*	↓*
18	Tryptophyl-Isoleucine	ESI+	C_17_H_23_N_3_O_3_	0.6438	244.591	318.1708073	↑**	↓*	↓*
19	7-Methylguanine	ESI-	C_6_H_7_N_5_O	0.9922	199.708	166.0710088	↑**	↓*	↓*
20	Estriol-16-Glucuronide	ESI-	C_24_H_32_O_9_	0.7058	316.8965	463.2011541	↑*	↓*	↓*

*P < 0.05, **P < 0.01.

### Biomarker identification and analysis of metabolic pathways

A module of MetaboAnalyst 5.0 was used for MetaboAnalyst pathway analysis. The levels of metabolites in intestinal contents were disturbed in rats with cerebral ischemia–reperfusion such as D-glutamine, D-glutamate metabolism, phenylalanine, tyrosine, and tryptophan biosynthesis, arginine biosynthesis, niacin and nicotinamide metabolism, purine metabolism, and sphingolipid metabolism. With the exception of purine metabolism and sphingolipid metabolism, treatment with *G. elata* Blume significantly reversed the changes in these pathways induced by CIRI (**Figures 10B, C**).

### Spearman correlation analysis

**FIGURE 11:**
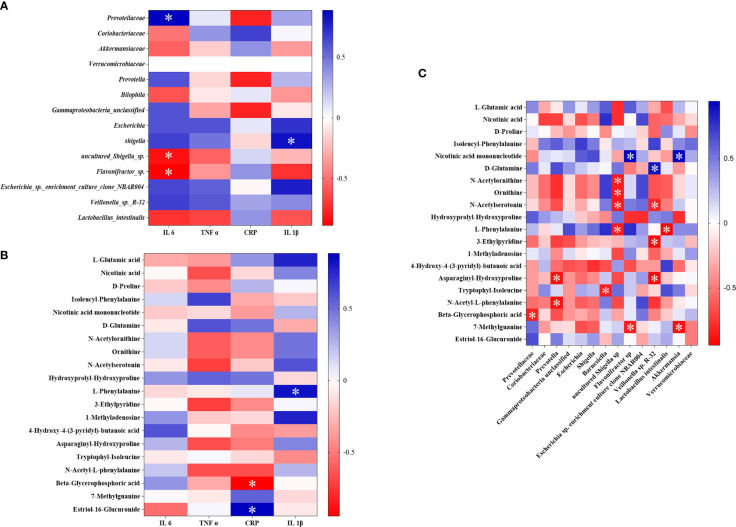
The association heat map of correlation for reversed intestinal flora and inflammatory factors **(A)**, potential biomarkers and inflammatory factors **(B)**, and intestinal flora and potential biomarkers **(C)**. *P < 0.05.

In order to evaluate the interaction among intestinal flora, metabolites, and inflammation, Spearman correlation analysis was performed on the 14 different flora, 20 different biomarkers, and the detected inflammatory factors that were found in the above study between the SG and CIRI (**Figures 11A, B**). After Spearman correlation coefficient analysis, a conspicuous dependence was found between some inflammatory factors and specific intestinal flora or potential biomarkers (*P* < 0.05). At the same time, it shows that there are also some significant correlations between intestinal flora and biomarkers (*P* < 0.05) (**Figure 11C**).

It was discovered that *Prevotellaceae* was significantly positively correlated with IL-6, and *Lactobacillus intestinalis* was negatively interrelated to IL-1β. After administration of *G. elata* Blume, *Prevotellaceae* was significantly downregulated and *Lactobacillus intestinalis* was significantly upregulated. L-phenylalanine was significantly positively interrelated to IL-1β, and beta-glycerophosphoric acid was significantly negatively interrelated to hs-CRP. After administration of *G. elata* Blume, L-phenylalanine was downregulated and beta-glycerophosphoric acid was upregulated. There were also significant negative correlations between L-phenylalanine and *Lactobacillus intestinalis* and between beta-glycerophosphoric acid and *Prevotellaceae*.

## Discussion

### Analysis of intestinal flora

In the IG 8d and IG 14d groups, beneficial bacteria including *Verrucomicrobiaceae*, *Lactobacillus*, and *Akkermansiaceae* were significantly higher than those in the CIRI group. Pathogenic bacteria such as *Escherichia*, *Shigella*, and the potential pathogenic bacteria *Prevotella* were present at conspicuously lower levels in the IG 8d and IG 14d groups than those in the CIRI group. These results indicated that *G. elata* Blume regulated the structure of intestinal flora and protected against CIRI by increasing the abundance of bacteria that are beneficial for the host, reducing the abundance of bacteria that are harmful.

*Prevotella* is a potentially pathogenic bacterium with pro-inflammatory properties (Iljazovic et al., 2021). *Prevotellaceae* is also related to inflammation (Liu N. et al., 2021). *Escherichia* is a pathogenic bacterium; increased *Escherichia* can promote intestinal inflammation (Guo et al., 2021). *Akkermansia* belongs to *Verrucomicrobia*, which is negatively correlated with obesity, diabetes, inflammation, and disturbance of the intestinal flora. *Akkermansia* can regulate intestinal immunity and promote intestinal health (Fuerst, 2019), which are important in maintaining intestinal integrity and fighting infection. *Lactobacillus* can metabolize a large number of SCFAs to regulate intestinal immune and barrier functions, thereby improving intestinal permeability, inhibiting inflammation, and reducing contents of IL-6, IL-8, and TNF-α in serum (Waitayangkoon et al., 2020).

Studies have shown that the expression of IL-6 and hs-CRP is upregulated after ischemic stroke, which are associated with intestinal flora disorders (Chen et al., 2021; Pang et al., 2021; Wang et al., 2022). An imbalance in intestinal flora can lead to increased phenylalanine and isoleucine in the peripheral accumulation, which can promote inflammation (Wang et al., 2019). Disturbance of the intestinal flora is associated with the severity of ischemic stroke, and it also may be closely related to metabolism and inflammation in the host (Xian et al., 2022). It was discovered in this study that *Prevotellaceae* was significantly positively correlated with IL-6; *Lactobacillus intestinalis* was negative interrelated to IL-1β and L-phenylalanine. *Prevotellaceae* and *Lactobacillus intestinalis* showed a significant trend of callback after administration of *G. elata* Blume.

### Metabolomics analysis

Amino acid metabolism is closely related to ischemic stroke, and metabolism of glutamine, arginine, lysine, and phenylalanine is particularly important in the acute phase post-stroke. A disorder of tryptophan metabolism (Yoon et al., 2022), imbalance of circulation between glutamic acid and glutamine (Sidorov et al., 2020), and rise of phenylalanine (Khan et al., 2020) and arginine levels (Grosse et al., 2020) have been observed in the acute phase of acute ischemic stroke, which indicated that the changes in these amino acids are markers of the acute phase post-stroke. In this research, the levels of N-acetylserotonin, D-glutamine, and L-phenylalanine were increased in the feces of CIRI rats. Furthermore, the arginine biosynthesis pathway was significantly enriched in CIRI. These metabolites were recovered to the levels observed in the SG to varying degrees after *G. elata* Blume administration. These results indicated that *G. elata* Blume may improve CIRI by regulating tryptophan metabolism, balancing the levels of glutamate and glutamine, and reducing the levels of phenylalanine and arginine.

Excessive inflammatory responses occur during acute cerebral ischemia, especially during reperfusion (Zheng et al., 2019). Some amino acid metabolic pathways are involved in inflammatory responses. A study has shown that some amino acids and their metabolites have the function of immune regulation (Leppkes and Neurath, 2020). Cytokines affect amino acid metabolism by affecting the transport, perception, and regulation of amino acids and the enzymes related to amino acid metabolism. In contrast, amino acids or their metabolites can affect the expression of cytokines by regulating the activation and phenotypes of immune cells.

Inflammatory cytokines such as TNF-α, IL-2, and IL-6 can upregulate the activities of arginine and indoleamine 2,3-dioxygenase (IDO), promoting the metabolism of arginine and tryptophan. Studies have reported that inflammatory cytokines could upregulate arginase activity and promote arginine metabolism (Qin et al., 2012; Baek et al., 2020; Haoran et al., 2020; Chen et al., 2022). These findings may explain why arginine levels were not increased in the CIRI group. Glutamine levels are associated with the markers of systemic inflammation including CRP and IL-6 (Sirnio et al., 2019).

**Figure 12:**
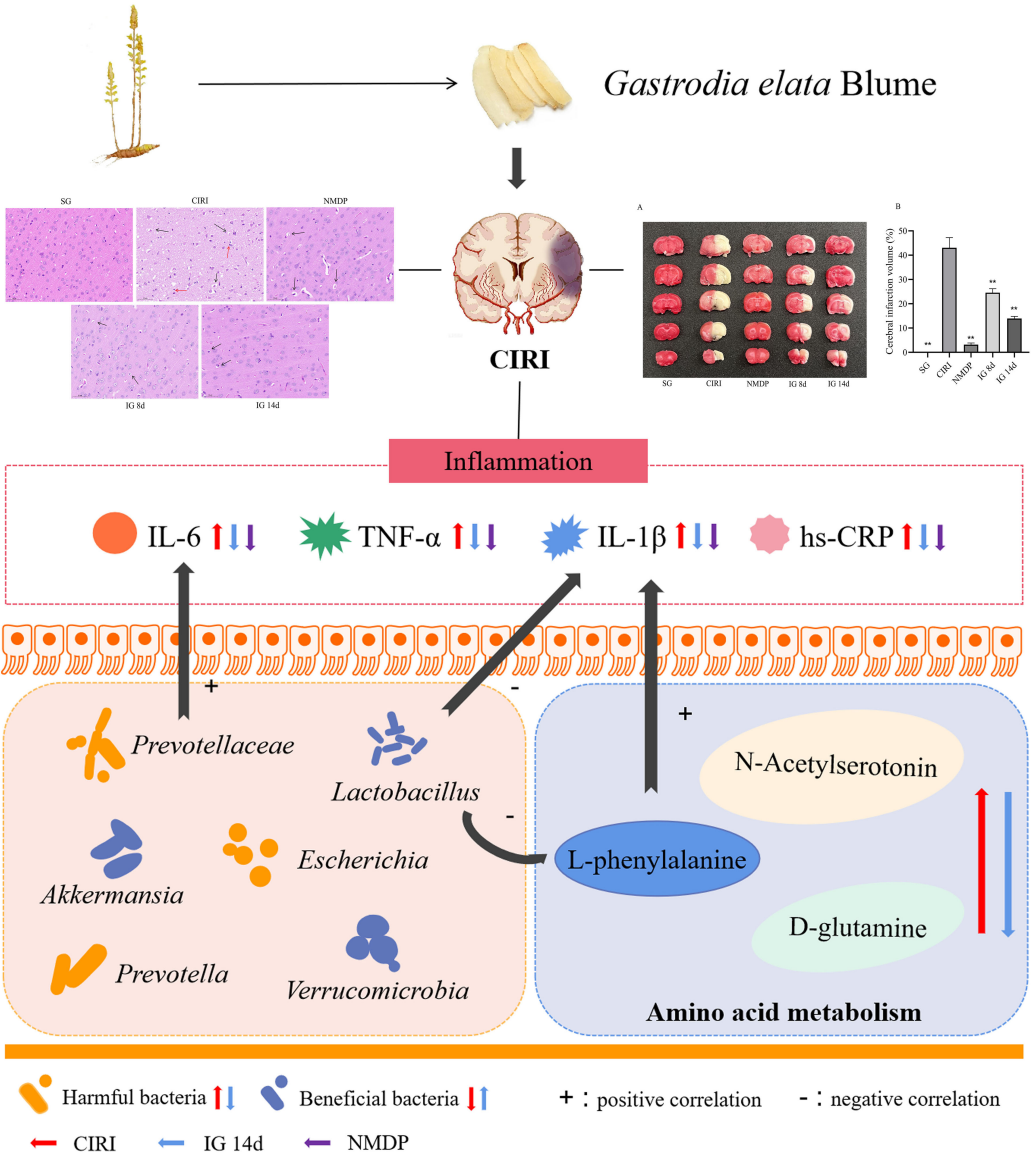
Mechanism diagram of Spearman correlation analysis. **P < 0.01.

The above study found that the structure of the intestinal flora was changed after *G. elata* Blume gavage in CIRI rats; these changes may affect the degradation of L-arginine and L-glutamic acid. There were significant correlations among metabolites such as L-phenylalanine and beta-glycerophosphoric acid, intestinal bacteria such as *Lactobacillus intestinalis* and *Prevotellaceae*, and inflammatory factors. *G. elata* Blume has the callback effect on these indexes in the treatment of CIRI. It indicates that changes in inflammatory factors that were found in the study may be regulated by amino acid metabolism and intestinal flora (**Figure 12**).

## Conclusion

This study showed that cerebral ischemia–reperfusion induced inflammation, disordered amino acid metabolism, and changes of intestinal microflora structure in rats. It was found that the inflammatory factor content, dysbiosis of the intestinal flora, and associated metabolic differences induced by CIRI were mitigated by *G. elata* Blume administration. These findings suggested that *G. elata* Blume may inhibit inflammation, regulate intestinal microflora structure, and ameliorate the disordered amino acid metabolism in CIRI rats. This may be related to the interaction among inflammatory factors, intestinal flora, and amino acid metabolism.

In this study, although *G. elata* Blume can regulate the intestinal flora structure of CIRI rats, it has not been explored in depth and there is a lack of intestinal flora transplantation verification. The intestinal flora of rats should be transplanted into CIRI rats after administration of *G. elata* Blume to observe whether it can treat CIRI, which may become a new breakthrough in the treatment of CIRI. It is expected that a large number of related studies will provide more basic and clinical research basis for *G. elata* Blume in regulating the intestinal flora to prevent and treat CIRI and a new perspective for CIRI clinical treatment drug intervention research. Taking intestinal flora as a treatment target can become a new idea for TCM in the treatment of cardiovascular and cerebrovascular diseases.

## Data availability statement

The data of 16S rDNA sequencing presented in the study are deposited in the Sequence Read Archive (SRA), accession number: SRP388582; The data of metabonomics presented in the study are deposited in the figshare: https://doi.org/10.6084/m9.figshare.21261294.

## Ethics statement 

The animal study was reviewed and approved by Committee on the Ethics of Animal Experiments of Anhui University of Chinese Medicine.

## Author contributions

XD, ZL, and YL designed the research, conducted performed the majority of the experiment, and revised the manuscript; BX, JC, JP, HY, and DW assisted on supported several experimental performances and deal with the statistical data; CJ and XW supervised the research and revised the manuscript. All authors contributed to the article and approved the submitted version.

## Acknowledgments

Authors would like to thank SHANGHAI BIOTREE BIOMEDICAL TECHNOLOGY CO., LTD and Heritage Base of TCM Processing Technology of NATCM of Anhui University of Chinese Medicine for supporting the work.

## Conflict of interest

The authors declare that this study received funding from Shanghai Biotree Biomedical Technology Co., Ltd. The funder had the following involvement in the study: instrumental detection of intestinal flora and intestinal contents.

## Publisher’s note

All claims expressed in this article are solely those of the authors and do not necessarily represent those of their affiliated organizations, or those of the publisher, the editors and the reviewers. Any product that may be evaluated in this article, or claim that may be made by its manufacturer, is not guaranteed or endorsed by the publisher.

## Supplementary material

Supplementary Figure 1 Images of brain by TTC staining **(A)**; Quantified data of TTC staining presented in ischemia volume (%) **(B)**. **P* < 0.05, ***P* < 0.01.

Supplementary Figure 2 PCA of QC and test samples. (**A**: PCA plot score in positive, the green dots stands for QC samples, the blue dots stands for test samples; **B**: One-dimensional distribution diagram of QC samples in positive mode; **C**: PCA plot score in negative mode, the green dots stands for QC samples, the blue dots stands for test samples; **D**: One-dimensional distribution diagram of QC samples in negative mode). (For interpretation of the references to color in this figure legend, the reader is referred to the web version of this article.)

Supplementary Figure 3 The correlation analysis of QC sample in positive and negative mode.

Supplementary Figure 4 Total ion chromatogram in SG, CIRI, NMDP, IG 8d and IG 14d samples in negative ion mode.

Supplementary Figure 5 Total ion chromatogram in SG, CIRI, NMDP, IG 8d and IG 14d samples in positive ion mode.

Supplementary Figure 6 The OPLS-DA score plot and OPLS-DA permutation plot in negative mode.

Supplementary Figure 7 The OPLS-DA score plot and OPLS-DA permutation plot in positive mode.

## Abbreviations

CIRI, cerebral ischemia–reperfusion injury; TCM, traditional Chinese medicine; KEGG, Kyoto Encyclopedia of Genes and Genomes; ELISA, enzyme-linked immunosorbent assay; QC, quality control; LC-OE-MS, liquid chromatography-orbitrap exploris-mass spectrometer; PCA, principal component analysis; OPLS-DA, orthogonal projection to latent structures squares-discriminant analysis; VIP, variable importance in the projection; HMDB, Human Metabolome Database; GC-MS, gas chromatography and mass spectroscopy; hs-CRP, high-sensitivity C-reactive protein; TNF, tumor necrosis factor; IL, interleukin; IG, intragastric; LPS, lipopolysaccharide; SCFAs, short-chain fatty acids.
