# Supplementary figures and images for: Comprehensive evaluation of the mechanism of Gastrodia elata Blume in ameliorating cerebral ischemia–reperfusion injury based on integrating fecal metabonomics and 16S rDNA sequencing
Source: Front Cell Infect Microbiol. 2022 Oct 27;12:1026627. doi: 10.3389/fcimb.2022.1026627 (PMC9648199; doi:10.3389/fcimb.2022.1026627)

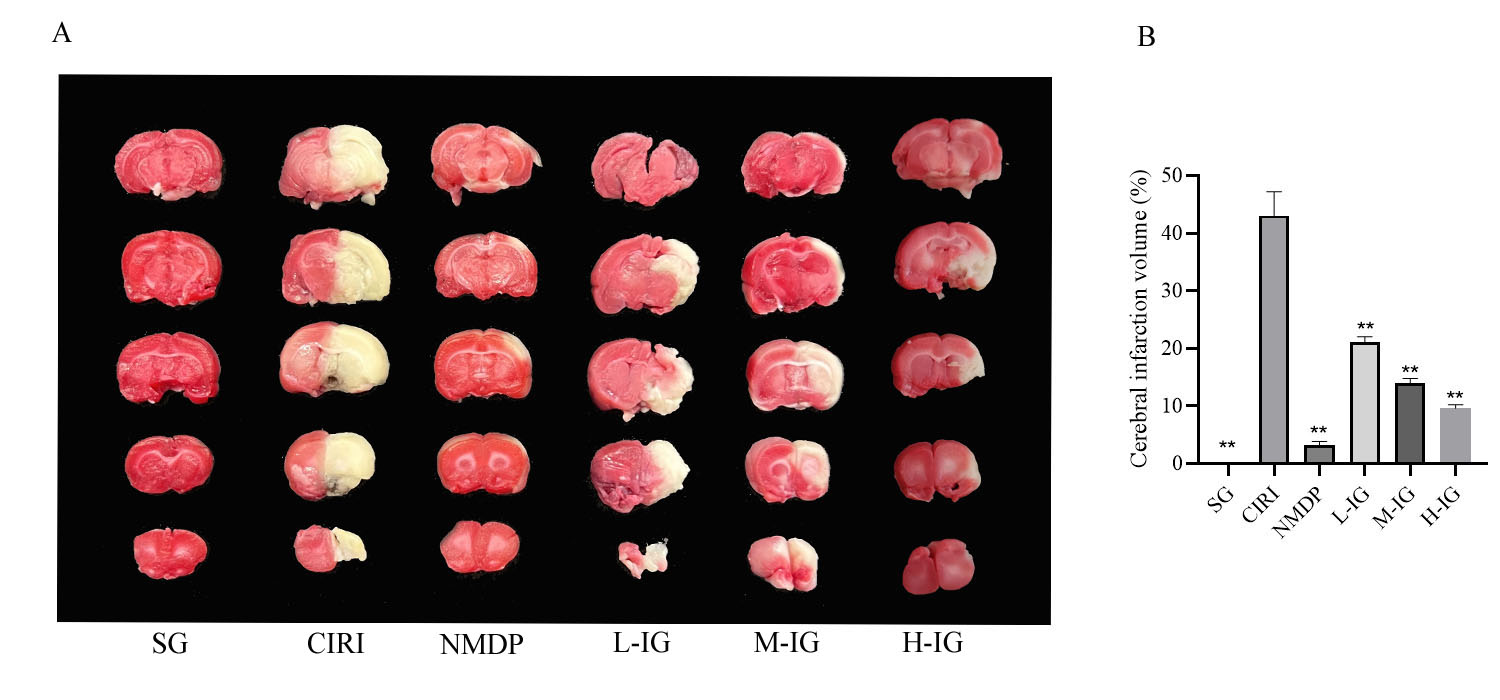

Supplement: Supplementary Figure 1 — Images of brain by TTC staining (A); Quantified data of TTC staining presented in ischemia volume (%) (B). *P < 0.05, **P < 0.01. [file Image_1.jpeg]

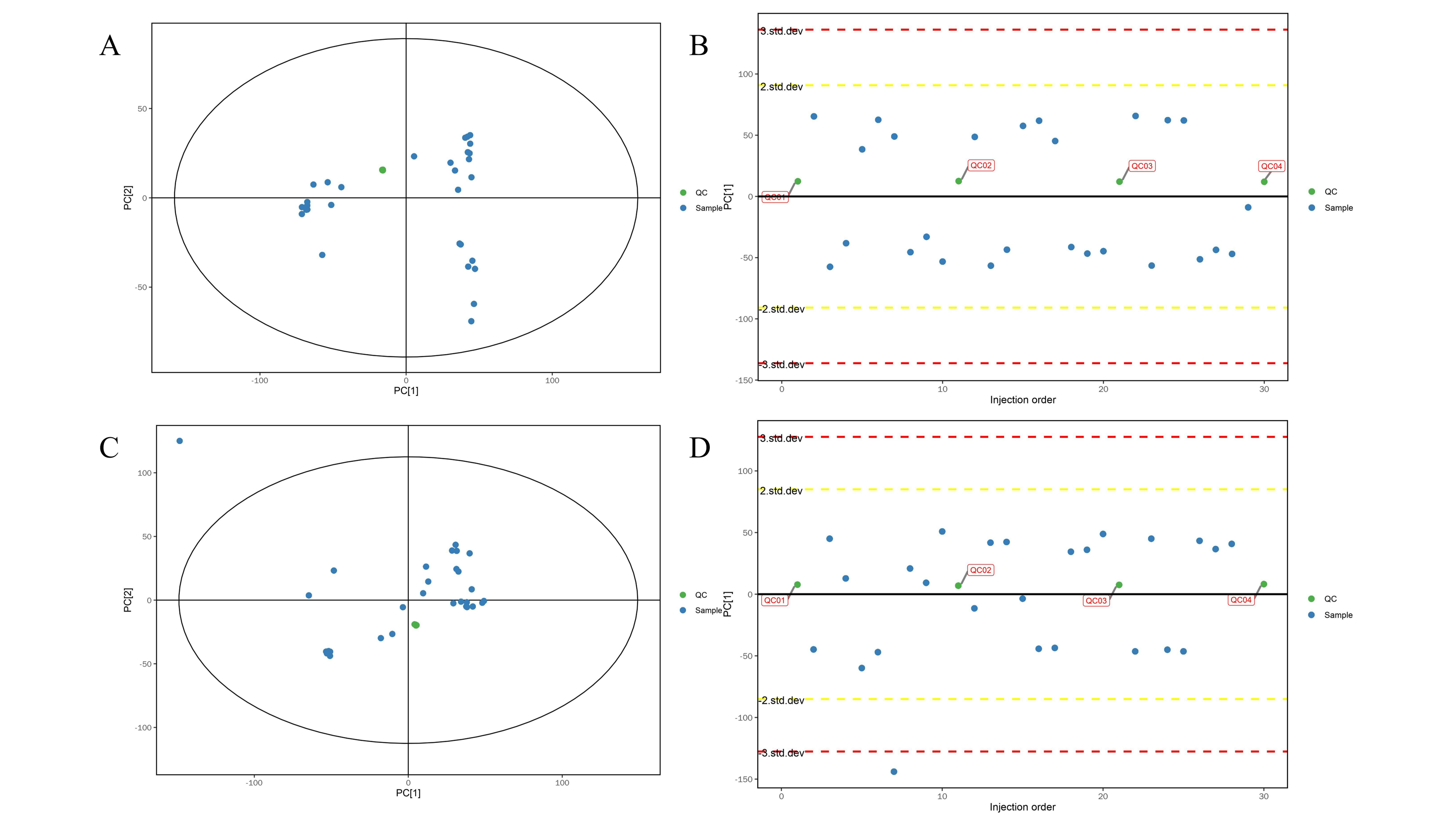

Supplement: Supplementary Figure 2 — PCA of QC and test samples. (A: PCA plot score in positive, the green dots stands for QC samples, the blue dots stands for test samples; B: One-dimensional distribution diagram of QC samples in positive mode; C: PCA plot score in negative mode, the green dots stands for QC samples, the blue dots stands for test samples; D: One-dimensional distribution diagram of QC samples in negative mode). (For interpretation of the references to color in this figure legend, the reader is referred to the web version of this article.) [file Image_2.jpeg]

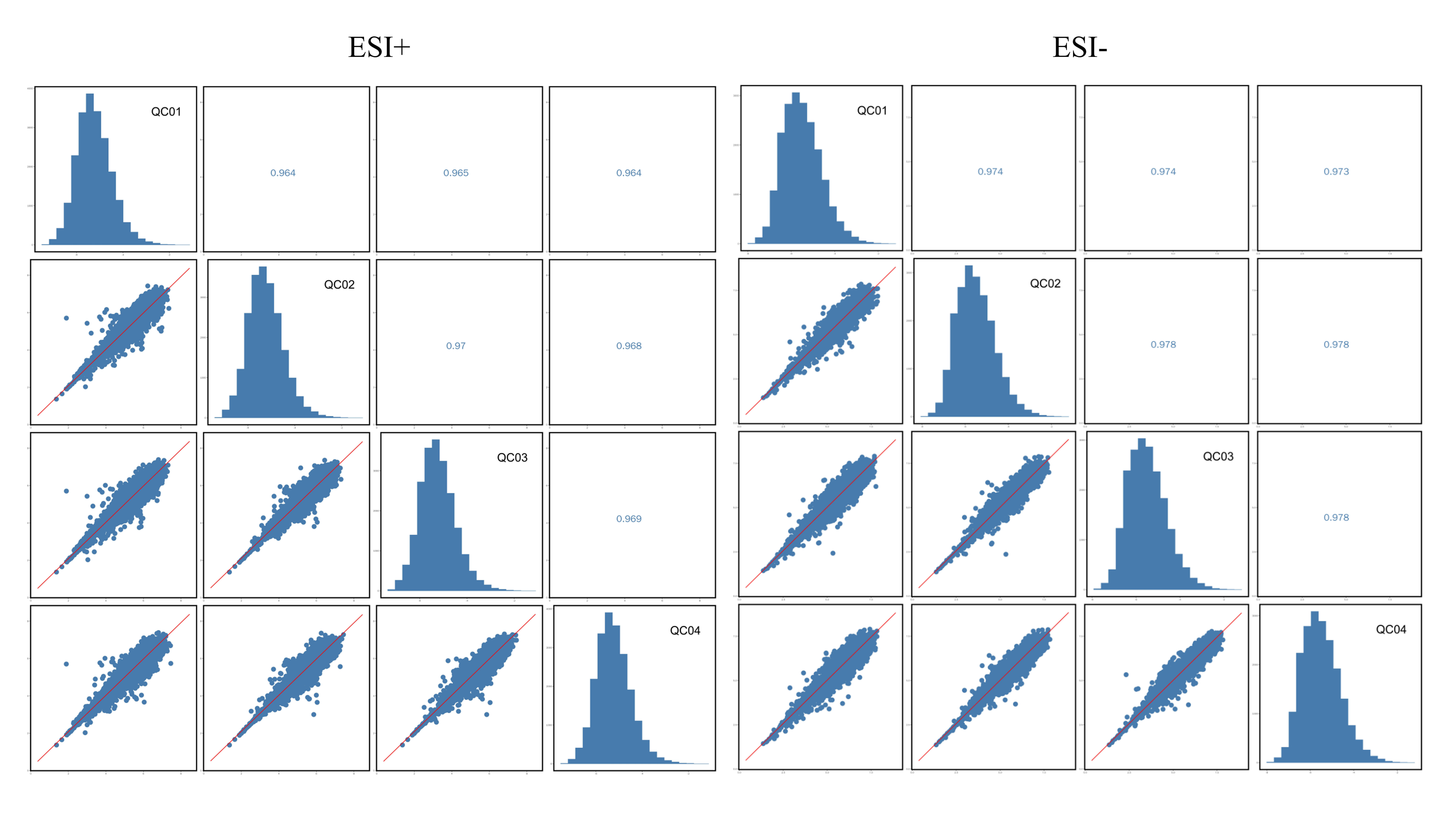

Supplement: Supplementary Figure 3 — The correlation analysis of QC sample in positive and negative mode. [file Image_3.jpeg]

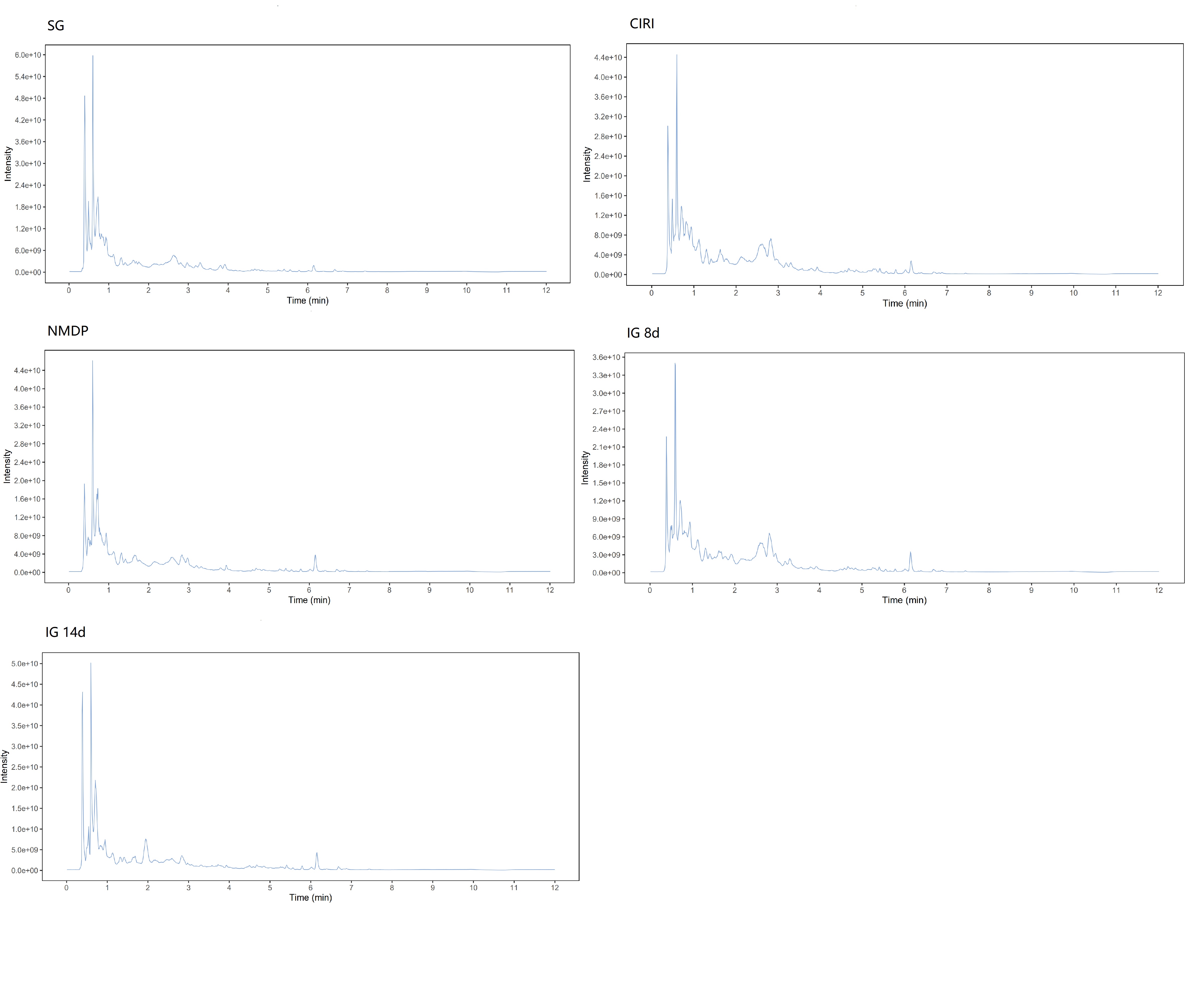

Supplement: Supplementary Figure 4 — Total ion chromatogram in SG, CIRI, NMDP, IG 8d and IG 14d samples in negative ion mode. [file Image_4.jpeg]

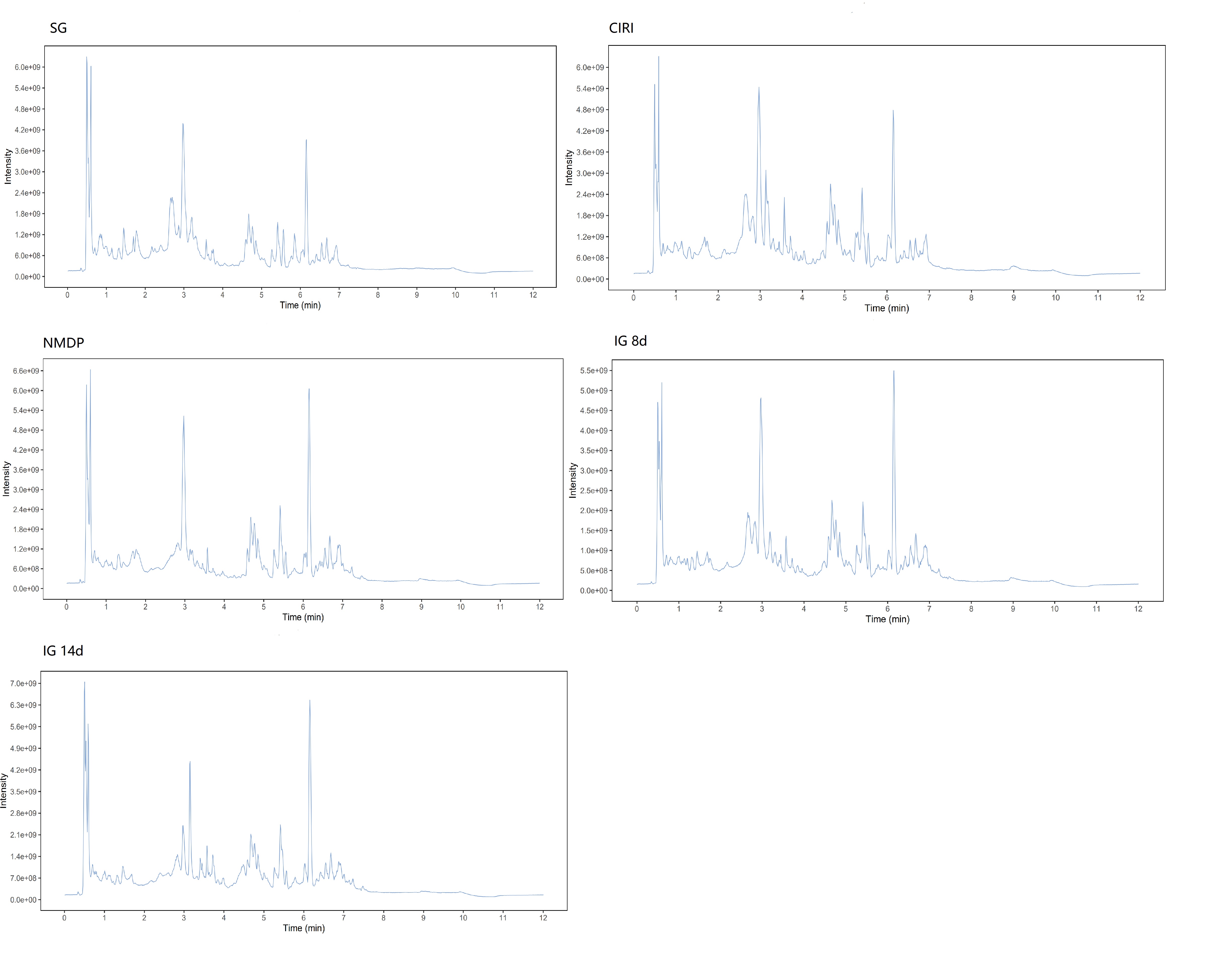

Supplement: Supplementary Figure 5 — Total ion chromatogram in SG, CIRI, NMDP, IG 8d and IG 14d samples in positive ion mode. [file Image_5.jpeg]

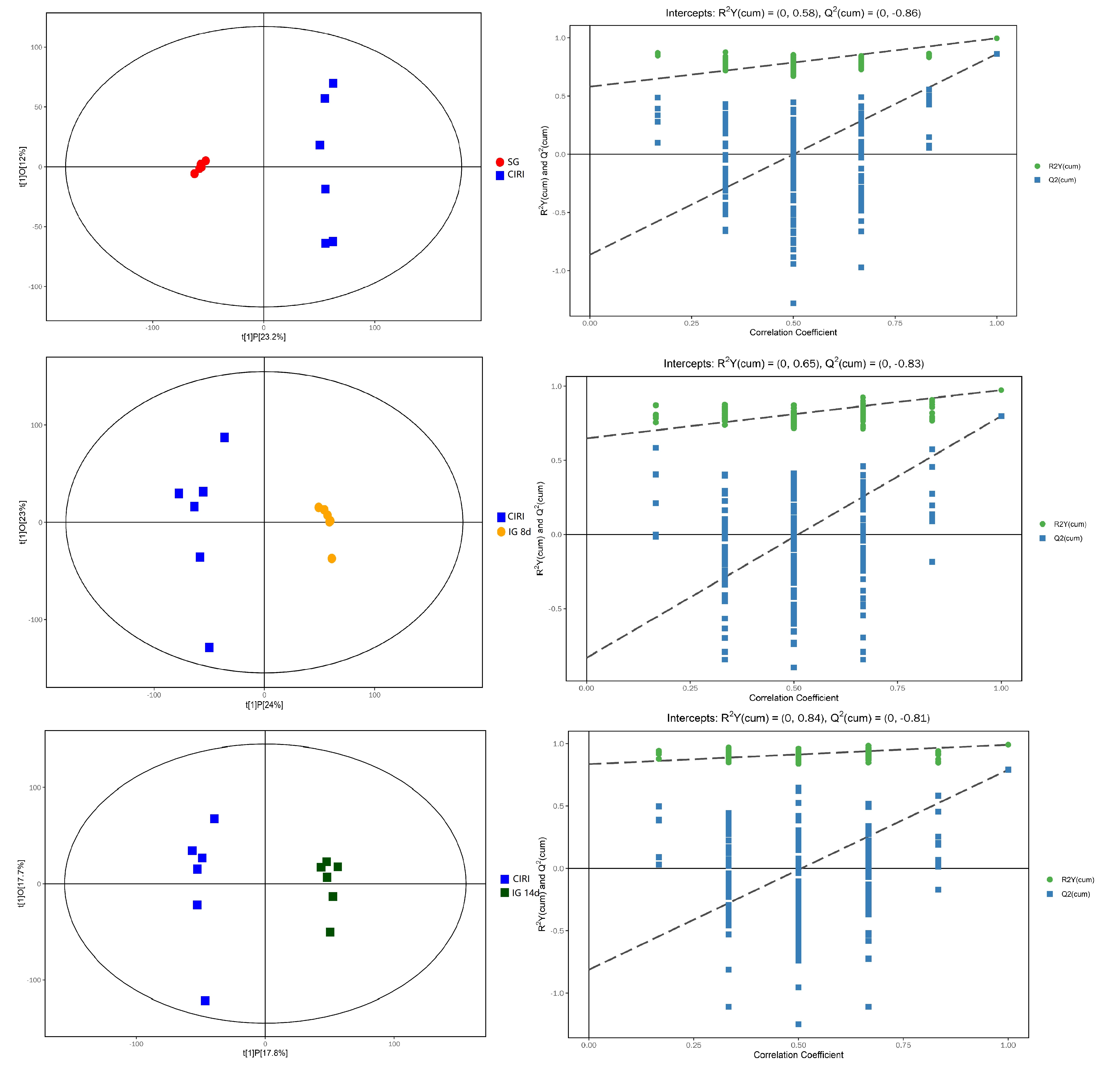

Supplement: Supplementary Figure 6 — The OPLS-DA score plot and OPLS-DA permutation plot in negative mode. [file Image_6.jpeg]

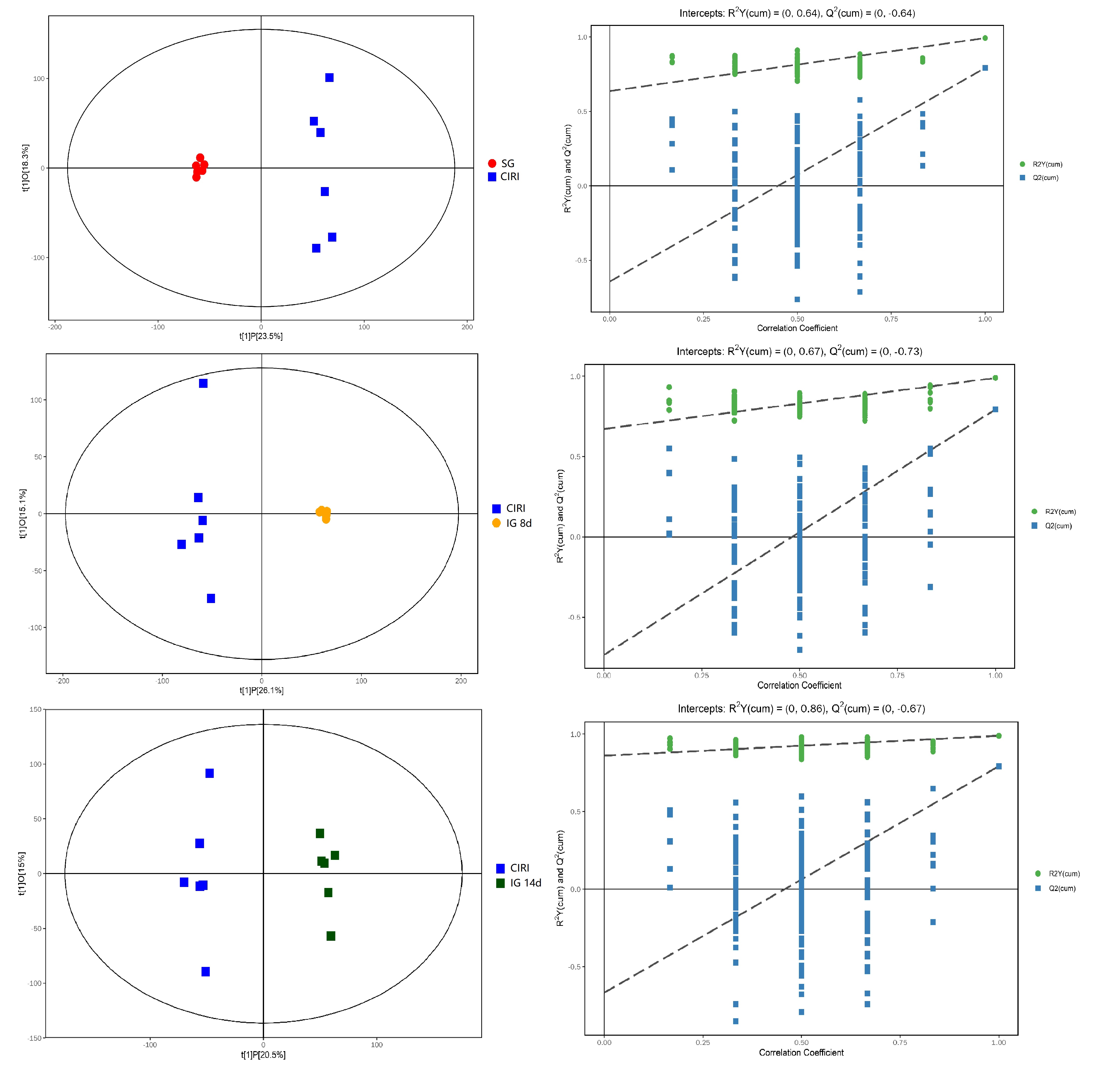

Supplement: Supplementary Figure 7 — The OPLS-DA score plot and OPLS-DA permutation plot in positive mode. [file Image_7.jpeg]
